# A New Approach for the Quantitative Evaluation of the Clock Drawing Test: Preliminary Results on Subjects with Parkinson's Disease

**DOI:** 10.1155/2010/283890

**Published:** 2010-06-29

**Authors:** Maria Francesca De Pandis, Manuela Galli, Sara Vimercati, Veronica Cimolin, Maria Vittoria De Angelis, Giorgio Albertini

**Affiliations:** ^1^“San Raffaele Cassino” Institute, San Raffaele SPA, 03043 Cassino (FR), Italy; ^2^Dipartimento di Bioingegneria, Politecnico di Milano, p.zza Leonardo Da Vinci 32, 20133 Milano, Italy; ^3^IRCCS “San Raffaele Pisana”, San Raffaele SPA, 00163 Roma, Italy

## Abstract

*Aims*. The realization of an experimental set-up for the quantitative and objective description of drawing using optoelectronic systems, which could be used when a quantification of the realization of specific drawing tests is required. *Methods*. Healthy subjects, subjects with Parkinson's Disease and subjects with Parkinson's Disease and Dementia were evaluated by the Mini Mental Scale Evaluation and by a new approach to the Clock Drawing Test, based on an optoelectronic acquisition. The new protocol hereby described aims to define a parameter related to the movement kinematics in the Clock Drawing test execution. *Results*. The experimental set-up revealed to be valid introducing new objective measurements beside the subjective Clock Drawing Test. This paper suggests the applicability of this protocol to other fields of motor and cognitive valuation, as well as the introduction of new parameters related to the graphic movement.

## 1. Introduction

Writing and drawing are the final output of a complex neurological, psychological, and motor action and can therefore be used to investigate both the movement capabilities and the cognitive functions of the subjects. 

Simple drawing tests are commonly used for the clinical evaluation of cognitive capabilities, especially in the elderly, in order to assess the presence of dementia and to estimate its extent.

Through the analysis of both the graphic gesture and the drawing contents, it is therefore possible to obtain a report of the subject's psicophisical health. In the cognitive deficits, the presence of different typologies of dementia (Alzheimer's disease AD, Mild Cognitive Impairment MCI, and Parkinson's disease dementia PDD) can be found. 

The Clock Drawing Test (CDT) is a qualitative neurological drawing test commonly used as a screening instrument for cognitive capabilities in the senile population and to evaluate the functional capabilities in the elderly.

The test is easily administrable, requires little time, and shows a good sensitivity in measuring the cognitive functions in the elderly. 

The CDT can be administered in two modalities: verbal command (*command condition*) and copying (*copy condition*). In the first one (*command condition*), the subject is asked to draw a clock with the clock hands indicating a particular time (ten minutes past eleven) [1, 2]. A well-drawn clock is supposed to have the circle, numbers from 1 to 12 in the correct order and position inside the circle, and the hands on the correct time.

It is also possible to give the subject a predrawn circle, in order to avoid that a badly drawn circle, or a too small one, influences the rest of the drawing [2–4]. 

The CDT *command condition* investigates the subject's language function (verbal comprehension) memory functions (recall of a visual engram, short-term storage, recall of time setting instructions), and executive function.

The test is highly sensitive for temporal lobe dysfunction (due to its heavy involvement in both memory and language processes), and frontal lobe dysfunction (due to its mediation of executive planning) [2].

In the second modality (*copy condition)*, the subject is given a printed clock, with the hands reading a certain time, and he is asked to replicate the drawing sideways, as accurately as possible.

This modality requires less use of language and memory functions but requires greater reliance on visual-spatial and perceptual processes. The copy condition is good for assessing parietal lobe lesions [2]; this region is responsible for stimuli recognition and for the recall of forms and structures. It processes the visual-spatial relationships and integrates proprioception with the other senses.

As the two modalities investigate different spheres of cognitive impairment, it is important to submit both the tests: a subject with a lesion of the temporal lobe would in fact copy correctly the predrawn clock but would result impaired in the command condition, for instance, by an incorrect spacing of the numbers or an incorrect representation of the clock face. On contrary a subject with a parietal lesion would adequately draw the clock in the command condition but would show pathologic features in the copy condition. 

In order to assure better sensitiveness multiscale scoring has been developed, which analyze both qualitative and quantitative characteristics of the drawing. These are the methods of Mendez, Cahn, and Freund which were adapted by the quantitative scoring evaluation system of Rouleau [1]. In particular, the Mendez system assigns a maximum of 20 points based on the presence of various characteristics of the clock, mostly related to the correct quantity and positioning of numbers and hands and to the absence of nonappropriate signs. According to the author healthy subjects do not miss more than two points, while subjects with Alzheimer's disease miss at least three. 

The test is commonly administered with the “pen and sheet” modality, with no use of computerized methods of acquisition, and the clinician visually does the evaluation and scoring. 

It is clear that this method involves great limitations in terms of measurement. First of all, a visual assessment is not completely inter- and intrarater reliable, because it is affected by the evaluator's experience and subjectivity. Secondly, it does not allow to memorize the temporal sequence of the graphic signs on the sheet. Every information about the temporal evolution of the graphic signs, such as reaction time and duration, is therefore lost, with a loss of important quantitative information. 

The most of the cognitive assessment is therefore still based on visual scoring systems, such as the ones described for the CDT (i.e., Mendez, Roleau scoring systems) or the widely used Mini Mental State Evaluation (MMSE).

In order to better quantify the gesture in the realization of CDT, we developed a preliminary method for the acquisition and analysis of cognitive drawing tests which allows to evaluate both the qualitative and quantitative features of the drawing.

The aims of this study are the following:

1. the development of an experimental setup for cognitive drawing tests, based on kinematic evaluation, which is suitable for pathological subjects,
2. the definition and computation of significant parameters related to the execution of the CDT,
3. the application of the experimental setup to a group of subjects with Parkinson's Disease (PD) and to a group of subjects with Parkinson's disease and dementia (PDD), in order to demonstrate the clinical applicability of the set up in patients with motor and cognitive impairment.

Clinical application is suitable for subjects with PD, as cognitive impairment and dementia have been highlighted as particularly common nonmotor complications in the case of Parkinson's Disease. 

Impaired cognitive domains in PDD include attention, memory, visual-spatial, constructional and executive functions [5, 6]. There are some indications from prospective studies that executive deficits may be the more important predictors of subsequent decline. However, the relationship between initial deficits and subsequent profile of dementia has not been clearly established [5].

Typically, drawing tests are used to assess constructional ability and praxis, either copying tasks or drawing common objects. As it was previously explained, construction and drawing tasks involve significant motor control and a range of cognitive functions. The contribution of motor dysfunction to such deficits has rarely been examined in PDD.

## 2. Materials and Methods

The subjects were recruited in the San Raffaele Parkinson's disease Centre of Cassino, FR, Italy and were diagnosed on the UK Brain Bank criteria. Healthy subjects were recruited between the patients' relatives. The study was approved by the Ethics Research Committee of the San Raffaele Pisana, Roma, Italy. All subjects were volunteers and gave informed consent to participation in the study. The subjects were evaluated with a standard clinical battery of tests. 

All the testing of PD patients was carried out during the ON phase, at their best motor condition, approximately 90 minutes after the first dose of levodopa, in the morning.

All patients underwent a quantitative assessment of their neurological condition using the Unified Parkinson's Disease Rating Scale (UPDRS). The scale is composed of six parts: mentation, behaviour, and mood (UPDRS I); activities of daily living (UPDRS II); motor examination (UPDRS III); complications of treatment (UPDRS IV); a global disability staging score (UPDRS V); a global activities of daily living score (UPDRS VI). The severity of the extrapyramidal symptoms was rated using the motor section of UPDRS (UPDRS III), the score for this part ranges from 0 to 108, and a higher score denotes greater disability. 

The functional status was assessed at the beginning and at the end of hospitalization by the Functional Independence Measure (FIM), which measures the overall functional disability. The scale includes 18 items, of which 13 are physical domains based on the Barthel Index (BI) and 5 items are cognition items. Each item is scored from 1 to 7 based on level of independence, where 1 represents total dependence. Possible scores range from 18 to 126, with higher scores indicating higher independence. The subjects underwent brain computed tomography and a complete neuropsychiatric evaluation. In order to summarize the cognitive conditions of the subjects, the Mini Mental State Evaluation (MMSE) and the Geriatric Depression Scale (GDS) were used for rating dementia and depression, respectively. According to the clinical MMSE scale a score inferior to 24/30 reveals the presence of dementia [4, 7]. GDS assigns a score of increasing depression level from 0 to 30.

Ten healthy subjects were evaluated in order to compose a control group (CG). Their mean age was 52.3 ± 11.7 years (age range from 52 to 73 years) and consisted of 6 females and 4 males. Then nine subjects with Parkinson's Disease (PD, mean  age = 63.6 + 9.5 years) and six subjects with PD and dementia (PDD, mean  age = 74.2 + 8.6 years) were evaluated. 

Data related to PD and PDD subjects are reported in Table 1. For every subject, the scores of the Unified Parkinson's Disease Rating Scale (UPDRS-III), the Functional Independence Measure (FIM), the Barthel Index (BI), the Mini Mental State Examination (MMSE), and the Geriatric Depression Scale (GDS) were detailed. 

The graphic gesture was acquired with an optoelectronic system with six cameras (SMART, BTS, Italy), at a frequency of 120 Hz, and with an integrated video system (Vixta, BTS, Italy) for videorecording.

The optoelectronic system is an equipment able to measures the 3D coordinates (*X*, *Y*, *Z*) of reflective markers. The acquisition was obtained by using markers of diameter = 10 mm in the configurations described in what follows.

**Figure 1:**
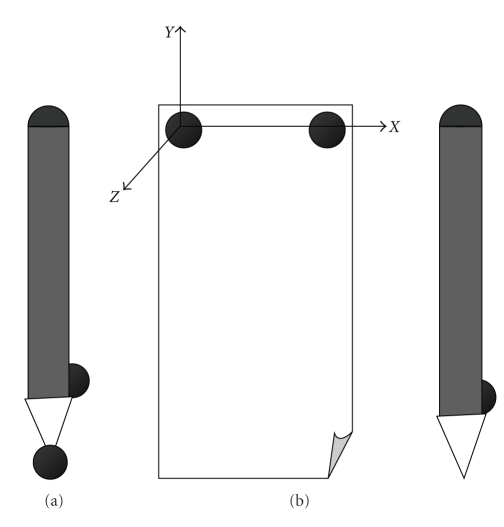
(a) Position of the markers for the static acquisition. (b) Position of the markers for the dynamic acquisition.

The first configuration, shown in Figure 1(a), was used for a static acquisition, in which the subject did not take part; the pen was laid on the table and the markers were acquired for five seconds, in order to calculate the position of the tip of the pen and allow the calculation of its position during the dynamic acquisition, in which the graphic test was executed by the subject. In the dynamic acquisition markers were positioned both on the sheet and on the pen (as shown in Figure 1(b)).

The CDT test was composed of two trials. In the first one (command condition), the subject was verbally asked to “draw a clock inside the predrawn circle and then draw the clock hands to indicate *ten minutes past eleven*.”

In the second task (*copy condition*), the subject was asked to copy, inside a printed circle, the clock shown on the paper.

Data reconstruction was carried out using the software Smart Tracking (BTS, S.p.a), which computes the tracking phase. After the tracking the acquisitions were computed using Smart Analyzer software (BTS, Italy, Version 1.10), which creates procedures ad hoc for the extraction of indexes of interest, angles, and trajectories. With this software, an algorithm was defined for the automatic calculation of the parameters of interest and for the realization of the related report. 

The *Trial duration*,  *T* *D*, [*s*] of the trial was computed using Smart Analyzer, in order to assess the reasoning and decision-taking time of PD and PDD subjects. 

**Figure 2:**
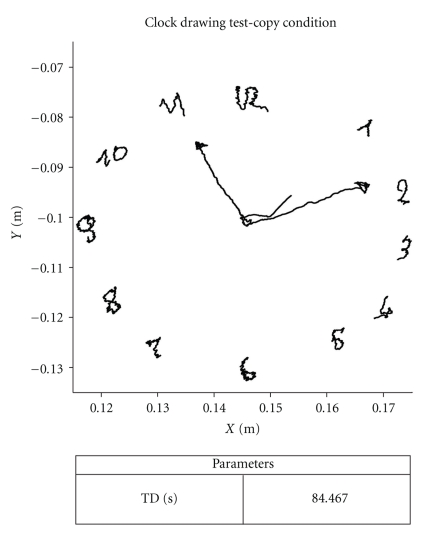
An example report of the Clock Drawing Test obtained with Smart Analyzer software, drawn by a CG subject.

An example of the layout of the test, obtained with Smart Analyzer software, is shown in Figure 2.

### 2.1. Statistical Analysis

Data were grouped by three categories: healthy control group subjects (CG), subjects with Parkinson's Disease (PD), and subjects with Parkinson's Disease Dementia (PDD). PD and PDD subjects were both evaluated in the on-medication phase (on levodopa treatment). For each group the mean and standard deviations were calculated.

In order to give statistical validity to the chosen parameters, some statistic tests were done with the use of Statistica software (Version 7.0). For every test, the *P*-value was taken into account, its significance threshold being  .05, so that values minor than the threshold were considered statistically significant.

First a normality test (Lilliefors and Kolmogorov-Smirnov tests) was done to verify the kind of distribution of the parameters. Comparison between independent samples (i.e., PD versus PDD) was assessed with the Mann-Whitney *U*-Test. Partial correlation analysis was used to determine whether the correlation between the variables of interest still held once controlling for a third “confounding” variable.

## 3. Results and Discussion

Statistical analysis of the duration index revealed differences between PD and PDD subjects in both command and copy conditions. The means and standard deviations of trial duration, for the two trials and the three groups, are detailed in Table 2. 

The CG subjects draw with comparable durations for the two tasks. No statistically significant difference was found, for duration, between CG and PD subjects, while there was difference between CG and PDD and between PD and PDD.

Performances were slower for both PD and PDD groups in the command condition. It can be noticed that, for both command and copy conditions, PD draw faster than PDD. 

**Figure 3:**
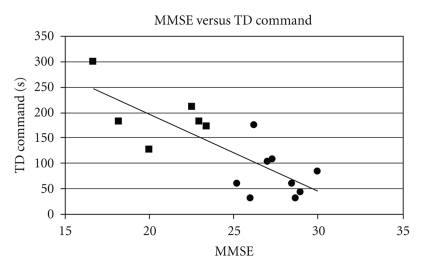
Relation between the MMSE scoring and duration in the CDT command condition (*r* = −0.75). Squares are PDD, circles are PD.

**Figure 4:**
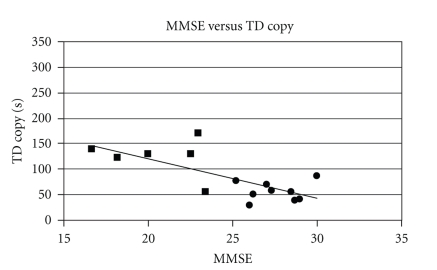
Relation between the MMSE scoring and duration in the CDT copy condition (*r* = − 0.81). Squares are PDD, circles are PD.

TD is a temporal parameter, and it can be therefore influenced by the presence of motor deficits such as bradykinesia, which are characteristic of Parkinson's Disease. In order to evaluate the validity of the trial duration (TD index) in the assessment of cognitive function, we calculated the partial correlation between MMSE and duration for the whole PD and PDD subjects, while controlling for the UPDRS III scores. The degree of motor impairment, in fact, could act as a confounding variable and therefore impact on the correlation between the variables of interest (MMSE and TD). The partial correlation between MMSE and TD was strong (command condition *r* = −0.75; copy condition *r* = −0.81) when controlling for motor impairment, meaning that UPDRS III is not influencing the correlation. In Figures 3 and 4, the statistically significant correlation between the MMSE scoring and TD for both drawing conditions is shown; the correlation is negative, meaning that lower MMSE (therefore higher degrees of cognitive dysfunction) led to higher reasoning time and thus to higher duration of the trials. 

The correlation was higher in the command condition trial, meaning that a stronger relation occurred between degree of dementia and duration of the trial in the command condition. This result can be interpreted in the light of the characteristics of PDD dysfunctions: the command condition, in fact, studies the impairments related to the frontal lobe, where executive function is processed. As this function generally appears to be the most compromised in PDD, we expected a higher difficulty in the command condition task, and thus higher durations of the trials (longer decision taking and executive time).

## 4. Conclusions

In this study, an experimental setup was defined for the quantitative and objective assessment of cognitive drawing tests. This setup was firstly applied to a group of healthy subjects (CG), then to a group of subjects with Parkinson's Disease (PD), and to a group of subjects with Parkinson's Disease and dementia (PDD), to assess the validity of the setup in the clinical usage.

The graphic gesture was acquired with an optoelectronic system with six cameras. Markers were put on the pen and on the sheet.

The subjects were asked to execute the Clock Drawing Test (CDT), a commonly used clinical test for the assessment of cognitive dysfunction. The test was administered in two different modalities: the command and the copy conditions. Each of these investigates specific cerebral areas and thus is sensitive to differently located brain damages [2]. 

Rather than concentrating on the CDT scoring itself, our aim was to develop a quantitative measurement. The CDT, in fact, has always been visually rated by the clinician, while only a few attempts were made to introduce objective and quantitative parameters [3].

We analyzed duration of the trials (Trial Duration, TD) for the two conditions of test. We found, indeed, that duration of the trial can be a useful, objective parameter for the assessment of cognitive performance. 

The calculation of the partial correlation between MMSE scores and duration of the trials, with control of the UPDRS III variable, revealed that motor impairment did not significantly impact on the correlation between trial duration and MMSE; the strong negative correlation showed that a higher degree of dementia led to longer durations of the trials, presumably because the impaired subjects had more reasoning difficulties. In particular, PDD subjects appeared to be more compromised in terms of executive function, which is investigated by the CDT command condition. Both PD and PDD performed faster in the copy condition, but still there was statistically significant difference between the two groups in both the trials. As the two conditions investigate different cognitive areas, we can suggest that the cognitive difficulty was less severe in the copy condition.

All trials revealed to be well tolerated by the subjects, who were able to complete the requested tasks without excessive fatigue. 

The optoelectronic system revealed to be advantageous both for a quantitative measurement of the parameters and for being a practical, noninvasive technique; it allowed the subjects to freely draw, recreating the “pen and sheet” condition and thus allowing natural movements. 

The test was completely not invasive and did not limit in any way the subject's motion. The application of the markers on the pen and the sheet did not require a specific competence, as it would have been, for instance, in the case of the application of an anatomic protocol, and was carried out in a few seconds.

The protocol was developed using Smart Analyzer software and, starting from the acquisition of the gesture with the optoelectronic system, allowed everyone to compute the data automatically, using the related protocol.

The protocol permits an easy employment in clinic analysis, thanks to the simple movements requested, the poor number of trails, the little duration, the complete noninvasive and natural conditions of testing. These characteristics suggest the applicability of this protocol with regard not only to Parkinson's Disease but also to other fields of motor and cognitive evaluation. Other parameters, such as drawing velocity or drawing sequence, could be evaluated. Besides, the protocol could be employed for the quantitative characterization of other graphic movements (including writing), particularly suitable for clinical applications.

## Figures and Tables

**Table 1:** Group of PD and PDD subjects.

Diagnosis	Age (years)	Gender	Years of PD	UPDRS in ON	Hoehn & Yahr in ON	FIM in ON	BI in ON	MMSE	GDS
PDD	62.0	f	2.0	37.0	2.5	84.0	72.0	18.3	13
PDD	66.0	f	10.0	20.0	3.0	79.0	63.0	18.2	11
PDD	75.0	m	5.0	32.0	3.0	85.0	73	20.0	13
PDD	77.0	f	19.0	49.0	3.0	56.0	49.0	16.7	11
PDD	81.0	f	9.0	44.0	3.0	77.0	71.0	22.5	15
PDD	84.0	f	8.0	36.0	3.0	82.0	64.0	23.4	7
PD	43.0	f	4.0	21.0	2.0	98.0	76.0	30.0	13
PD	57.0	m	10.0	26.0	2.0	99.0	79.0	28.7	11
PD	61.0	f	4.0	36.0	3.0	88.0	56.0	28.5	13
PD	63.0	f	20.0	21.0	3.0	92.0	84	29.0	10
PD	65.0	f	4.0	34.0	2.5	99	90	26.2	8
PD	67.0	f	8.0	36.0	3.0	98.0	30.0	25.2	11
PD	69.0	f	6.0	47.0	3.0	87.0	69	26.0	15
PD	71.0	f	6.0	30.0	3.0	79.0	70.0	27.3	11
PD	76.0	m	6.0	22.0	3.0	81.0	72.0	27.0	12

**Table 2:** Trial duration (mean and standard deviations) for the three groups and for both drawing conditions.

Trial Duration (TD) [s]						
	CDT Command			CDT Copy		
	Mean	St Dev	*P*-value	Mean	St Dev	*P*-value

CG	44.6	14.1		44.1	11.7	
PD	77.4	46.4	∗ ♦	55.7	18.8	∗ ♦
PDD	195.9	57.1		124.2	37.3	

*: *P*-value <.05 for PD versus PDD; ♦: *P*-value <.05 for CG versus PDD; ●: *P*-value <.05 for CG versus PD.
